# The Small Tellurium Compound AS101 Ameliorates Rat Crescentic Glomerulonephritis: Association with Inhibition of Macrophage Caspase-1 Activity *via* Very Late Antigen-4 Inactivation

**DOI:** 10.3389/fimmu.2017.00240

**Published:** 2017-03-07

**Authors:** Yafit Hachmo, Yona Kalechman, Itai Skornick, Uzi Gafter, Rachel R. Caspi, Benjamin Sredni

**Affiliations:** ^1^C.A.I.R. Institute, The Safdiè AIDS and Immunology Research Center, The Mina & Everard Goodman Faculty of Life Sciences, Bar-Ilan University, Ramat Gan, Israel; ^2^Laboratory of Nephrology and Transplant Immunology, Rabin Medical Center, Petah-Tikva, Israel; ^3^Tel Aviv University, Tel Aviv, Israel; ^4^Laboratory of Immunology, National Eye Institute, National Institutes of Health, Bethesda, MD, USA

**Keywords:** glomerulonephritis, cytokines, integrin, caspase-1, AS101, inflammation

## Abstract

Crescentic glomerulonephritis (CGN) is the most aggressive form of GN and, if untreated, patients can progress to end-stage renal failure within weeks of presentation. The α4β1 integrin very late antigen-4 (VLA-4) is an adhesion molecule of fundamental importance to the recruitment of leukocytes in inflammation. We addressed the role of VLA-4 in mediating progressive renal injury in a rat model of CGN using a small tellurium compound. AS101 [ammonium trichloro(dioxoethylene-*o,o*′)tellurate]. This compound has been previously shown to uniquely inhibit VLA-4 activity by redox inactivation of adjacent thiols in the exofacial domain of VLA-4. The study shows that administration of AS101 either before or after glomerular basement membrane anti-serum injection ameliorates crescent formation or preserves renal function. This was associated with profound inhibition of critical inflammatory mediators, accompanied by decreased glomerular infiltration of macrophages. Mechanistic studies demonstrated vla-4 inactivation on glomerular macrophages both *in vitro* and *in vivo* as well as inhibition of caspase-1 activity. Importantly, this cysteine protease activity modification was dependent on VLA-4 inactivation and was associated with the anti-inflammatory activity of AS101. We propose that inactivation of macrophage VLA-4 by AS101 *in vivo* results in a decrease of inflammatory cytokines and chemokines produced in the glomeruli of diseased rats, resulting in decreased further macrophage recruitment and decreased extracellular matrix expansion. Thus, AS101, which is currently in clinical trials for other indications, might be beneficial for treatment of CGN.

## Introduction

The hallmark of human anti-glomerular basement membrane (GBM) disease is the production of autoantibodies targeting the non-collagenous (NC1) domain of the α3 chain of type IV collagen found in the GBM (1) leading to crescent formation and crescentic glomerulonephritis (CGN). CGN is the most aggressive form of glomerular inflammation, which presents clinically as rapidly progressive glomerulonephritis. It is characterized by disruption of the GBM, which leads to infiltration and proliferation of inflammatory cells such as macrophages in Bowman’s space (2).

The glomerulonephritis is associated with interstitial nephritis in many patients with anti-GBM disease and in animal models of CGN as well (3, 4). The inflammatory process gives way to glomerulosclerosis, interstitial fibrosis, tubular atrophy, and renal failure.

Although the pathogenesis of CGN is incompletely understood and likely involves several convergent pathways, there is general agreement that circulating mononuclear phagocytes play a central role. Administration of nephrotoxic serum to rodents results in a severe proliferative and necrotizing GN that is characterized by glomerular crescent formation and accumulation of leukocytes (2–4). It is thought that infiltrating cells release inflammatory mediators that influence the behavior of glomerular, tubular, and interstitial cells. This interaction between infiltrating and resident cells leads to cellular proliferation, matrix expansion, and may ultimately lead to glomerular sclerosis and interstitial fibrosis. Monocytes and macrophages appear to have a critical role, as ablation of macrophages in murine CGN reduced glomerular injury and improved renal function (5). Infiltrating glomerular macrophages are the major source of IL-1 (6) and tumor necrosis factor (TNF) (7). TNFα was shown to promote VCAM-1 and ICAM-1 glomerular expression and the recruitment of PMNs and lymphocytes were markedly reduced in TNF-deficient mice in experimental GN induced by anti-GBM antibody (8). Thus, strategies that reduce monocyte macrophage infiltration could be a promising avenue for complementary therapy of CGN.

Another strategy to prevent leukocyte infiltration in the kidney could be to interrupt the interaction between endothelial molecules involved in cell adhesion and their ligands on circulating inflammatory cells. The α4β1 integrin or very late antigen-4 (VLA-4) is expressed mainly on monocytes, lymphocytes, and eosinophils (9). AS101 is a potent *in vitro* and *in vivo* tellurium immunomodulator, with a wealth of potential therapeutic applications (10, 11). The compound is non-toxic and is currently in phase II/III studies in patients with cervical tumors and in phase I/II studies in patients with aging macular degeneration. From a mechanistic point of view, much of the biological activity of AS101 is directly related to its chemical redox interactions with vicinal thiols in the exofacial domain of VLA-4 (12), enabling the compound to mediate such diverse effects as abrogation of the acquired drug resistance of acute myelogenous leukemia (12) and amelioration of experimental autoimmune encephalomyelitis (13). The specific redox-modulating activities of AS101 result in a variety of beneficial biological effects in diverse preclinical and clinical studies (14). The anti-inflammatory properties were found crucial for the clinical activities of AS101, including the protective effects of AS101 in autoimmune diseases (15, 16) and in septic mice (17). The same thiol–redox interactions of AS101 enabled it to exert beneficial effects in a variety of tumor models in mice and humans where AS101 had clear antitumor effects (18, 19). Importantly, as part of its activity, AS101 exerts nephroprotective effects. It was able to reduce the level of immune complex deposition in the glomeruli, reduce proteinuria, prevent glomerular hypercellularity and mesangial expansion, and reduce the mean glomerular volume in a murine model of systemic lupus erythematosus (16) and prevent kidney damage in a murine model of septic peritonitis (17).

The anti-inflammatory properties of AS101 coupled with its unique mode of VLA-4 inactivation prompted us to evaluate its potential beneficial activities in CGN and determine the role of VLA-4 inactivation in these effects. In the present study, we demonstrate that administration of AS101 both before and after induction of CGN by αGBM injection ameliorates damage to the glomeruli and preserves renal function. This was associated with profound inhibition of inflammatory mediators and a decreased infiltration of macrophages. Mechanistic studies demonstrated that these effects were associated with vla-4 inactivation on macrophages and inhibition of caspase-1 activity in a VLA-4-dependent fashion. These data suggest that AS101 could have clinical potential as a therapeutic approach to glomerulonephritis.

## Materials and Methods

### Reagents

The reagents were as follows: fibronectin (FN) (Sigma; Rehovot, Israel) VCAM-1(R&D Biosystems, Minneapolis, MN, USA); BSA (Sigma); XTT cell proliferation kit (Biological Industries, Bet Haemek, Israel); FAM FLICA Caspase-3,7 and caspase-1 detection kit (Immunochemistry Technologies LLC, MN, USA); anti Cd49d, anti cd49e (Serotec, NC, USA); *Staphylococcus aureus* Cowan I (SAC) (Calbiochem-Behring Corp., La Jolla, CA, USA). LPS (Sigma); mouse anti-rat CD68 (ED1) (Serotec); rabbit anti-rat FN (Cedarlane, Burlington, ON, Canada); anti-mouse IL-18 (R&D Biosystems); anti-mouse IL-1β (Santa Cruz, Santa Cruz, CA, USA); anti αTubulin (Santa Cruz); anti-rat Thy-1.1(Cederlane); sheep anti-rat GBM (Probetex, San Antonio, TX, USA); rat IL-1β, IL-18, and TNFα ELISA kits (BiosourceThermo Fisher, Waltham, MA, USA); rat connective tissue growth factor (CTGF) and monocyte chemoattractant protein-1 (MCP-1) ELISA kits (MyBiosource, San Diego CA, USA). Creatinine and albumin assay kits (Abcam, San Francisco, CA, USA); AS101 (supplied by M. Albeck, Bar-Ilan University, Ramat Gan, Israel).

### Animals

Male Sprague-Dawley rats (180–200 g) were housed in individual metabolic cages with unlimited access to food and water. Experiments conformed to approved institutional protocols and were approved by the Institutional Animal Care and Use Committee.

### Experimental CGN

Rats were presensitized on day −5 by a subcutaneous injection of 6.25 mg sheep IgG in 0.5 ml emulsion (1:1 v/v) with Freund’s complete adjuvant. Glomerulonephritis was induced on day 0 by a single intravenous injection of 105 mg/kg sheep anti-rat GBM (αGBM). Experimental groups were as follows: daily i.p. injection with PBS without αGBM administration (negative PBS control); daily injection with PBS of αGBM-induced rats (positive control) and three treatment groups of daily i.p. injections with AS101 (100 μg/rat): starting 1 day before, 3 days after, or 6 days after αGBM administration.

### Isolation of Glomeruli

Glomeruli were isolated from the renal cortex of rats using the differential sieving method (20). The purity of glomeruli was >95%.

### Isolation and Culture of Glomerular Mesangial Cells

Isolated glomeruli were cultured in Dulbecco’s modified Eagle’s medium containing 5.5 mM d-(+)-glucose; 20% fetal bovine serum, 100 μg/ml streptomycin, 100 μg/ml penicillin, and 2 mM l-glutamine, at 37°C in 5% CO_2_. After 10 days, the cells had the typical appearance of spindle-shaped bundles, and no polygonal endothelial or epithelial cells could be detected. Cells stained positive with a rabbit polyclonal anti-rat Thy-1.1 antibody (mesangial cell marker).

### Isolation and Culture of Glomerular Macrophages

Glomeruli were cultured in Eagle’s MEM, containing 10% FCS at 37°C in a 5% CO_2_/air atmosphere for 3 days. Glomeruli were removed by decanting the medium after vigorous agitation. Adherent macrophages were removed by incubation for 3 min with trypsin–versene solution. Cells harvested were used immediately. These cells were 90% positive for ED1.

### Western Blot Analysis

Isolated glomeruli were lysed with lysis buffer [1 M Tris (pH = 7.4), 1.5 M NaCl, 1% Triton-X, 10% glycerol, 50 mM EDTA (pH = 8), 0.1 M sodium vanadate, 0.1 M PMSF, 0.1% protease inhibitor cocktail]. Samples were boiled for 5 min, electrophoresed on 15% or SDS-PAGE, transferred to nitrocellulose, and immunoblotted with specific antibodies (IL1β, IL-18). Blots were developed using horseradish peroxidase-conjugated secondary Abs and the ECL detection system.

### Quantitation of Caspase Activity

Isolated glomerular extracts were prepared in Tris/acetate buffer (pH 7.5) at 30°C. The extracts were centrifuged at 12,000 × *g* for 10 min, and the supernatant was collected. A volume of supernatant equivalent to 100 μg of protein was assayed for caspase-1 or caspase-3 activity using the colorimetric caspase-1 and -3 assay kits.

### Quantitation of Cytokine Levels

IL-1β, TNF-α, CTGF, MCP-1, and IL-18 ELISA kits were used for the quantitative measurement of these cytokines either in rat sera or urine, in supernatants of cultured cells or in glomerular lysates.

### Attachment Assay for Evaluation of VLA-4 Activity

96-well plates were coated with 80 μL of FN, VCAM-1, or BSA. Cells were incubated in the wells for 1 h with or without AS101 and were washed three times. The attached cells were tested by the colorimetric XTT (2,3-bis[2-methoxy-4-nitro-S-sulfophenynl]H-tetrazolium-5-carboxanilide inner salt) assay at 450 nm.

### Renal Histology

Resected kidneys were cut by a coronal section through the mid portion of the kidney. One-half was fixed in 10% buffered formalin. Paraffin blocks were prepared, 3 μM sections were cut from each block and stained with hematoxylin-eosin and periodic acid Schiff stains for detection of crescent formation.

### Immunohistochemistry

For ED-1 staining, fixed paraffin embedded kidney sections were incubated for 1 h with mouse anti-ED-1 (1:100) followed by horse anti-mouse Biotin-conjugated (1:500) antibodies. After incubation with secondary antibodies, the sections were incubated with HRP-streptavidin followed by DAB for an additional, washed and exposed to hematoxylin. Glomerular macrophages were counted under light microscope. One hundred glomeruli were counted/sample. For FN staining, the sections were incubated with rabbit anti-rat FN (1:100) followed by horse anti-rabbit Biotin-conjugated (1:500) antibodies. The sections were then incubated with HRP-streptavidin followed by DAB, washed, and exposed to hematoxylin. FN expansion was scored in 100 glomeruli/sample as follows: grade 0—no staining; grade 1—staining occupying up to 25% of glomerular surface area; grade 2—staining occupying 25–50% of glomerular surface area; grade 3—staining occupying more than 50% of glomerular surface area.

### Fluorescence-Activated Cell Sorter (FACS) Analysis

Rats were treated with anti-GBM and PBS or with anti-GBM and AS101 (+3). At 2 weeks after anti-GBM administration, macrophages were isolated from glomeruli of rats and evaluated for VLA-4 expression by FACS analysis. VLA-4 expression on isolated glomerular macrophages was determined after incubation with FITC Mouse Anti-Rat CD49d (serotec) by FACS [FACStar plus (Becton Dickinson) flow cytometer] using The Flow Jo software.

### Statistical Analysis

Data are presented as mean ± SE. For comparisons between groups in the *in vivo* and *in vitro* studies, we used the one- or two-way ANOVA. Linear correlation analysis (Pearson correlation) was applied to determine the correlation and association between parameters. Two-tailed *p* < 0.05 was considered statistically significant. The software used for all statistical analysis was IBM SPSS Statistics 21.

## Results

### Administration of AS101 Ameliorates Crescent Formation and Preserves Renal Function in Experimental CGM

**Figure 1:**
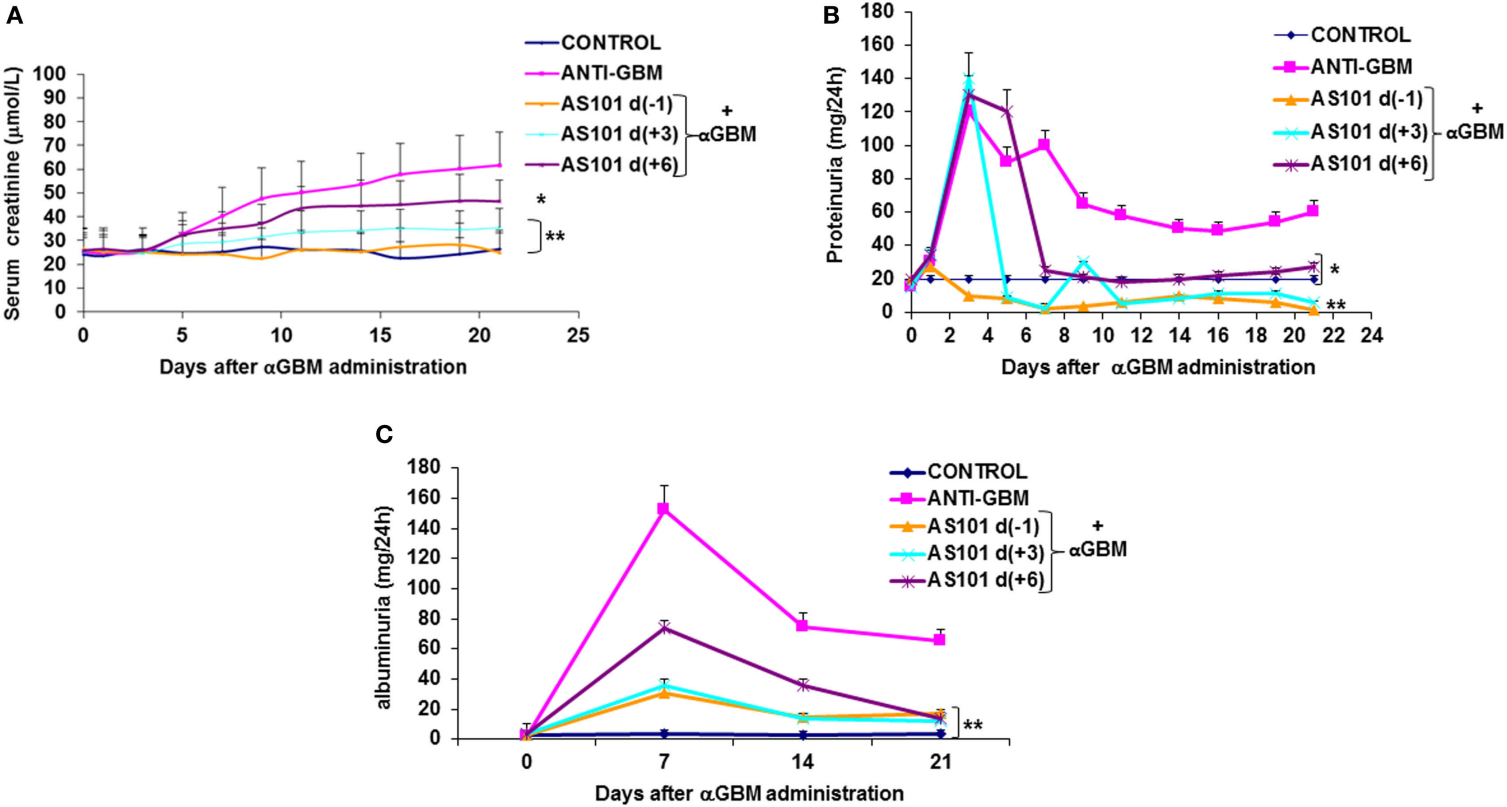
**Treatment with AS101 preserves kidney function in crescentic glomerulonephritis**. GN was induced by a single intravenous injection of αGBM [sheep anti-rat glomerular basement membrane (GBM)] in rats presensitized 5 days earlier with sheep IgG, as described in Section “Materials and Methods.” Experimental groups were as follows: daily i.p. injection with PBS without αGBM administration (negative control); daily injection with PBS of αGBM-induced rats (positive control) and three treatment groups of daily i.p. injections with AS101 (100 μg/rat) starting 1 day before (−1), 3 days after (+3), or 6 days after (+6) the αGBM administration. Serum creatinine **(A)** and proteinuria **(B)** were recorded on days 0, 1, 3, 5, 7, 9, 11, 14, 16, 19, and 21. Albuminuria **(C)** was recorded on days 0, 7, 14, and 21. Data are presented as mean ± SE of eight rats/group for each time point. *p < 0.05 decrease vs. αGBM. **p < 0.01 decrease vs. αGBM; the two-way ANOVA was used.

Rats were induced for CGM as described in Section “Materials and Methods.” Daily i.p. injections of AS101 (100 μg/rat) preserved renal function (Figure 1) as evidenced by decreased serum creatinine levels (Figure 1A) and decreased proteinuria (Figure 1B) and albuminuria (Figure 1C). These effects were marked and significant when treatment started 1 day before αGBM administration [AS101(−1)] or 3 days after αGBM administration [AS101(+3)]. Starting treatment 6 days after αGBM administration [AS101(+6)] still significantly improved renal function, although the effect was more modest.

**Figure 2:**
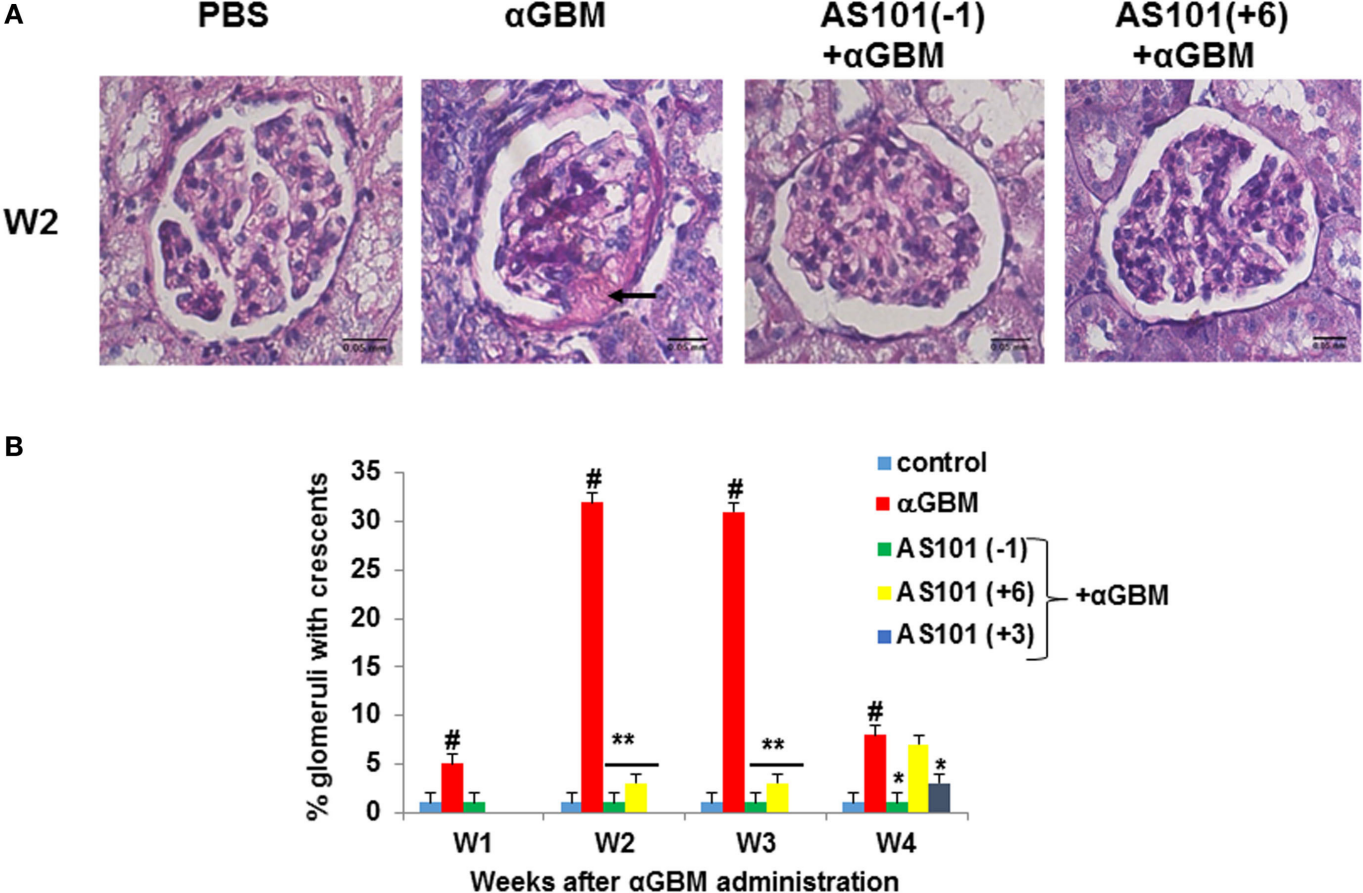
**Treatment with AS101 decreases crescent formation in crescentic glomerulonephritis**. Treatment protocol was as described in Figure 1 with one modification. Experimental groups were as follows: daily i.p. injection with PBS without αGBM administration (control); daily injection with PBS of αGBM-induced rats (αGBM only) and two treatment groups of αGBM-induced rats with daily i.p. injections of AS101 (100 μg/rat) starting 1 day before (–1), or 6 days after (+6) the αGBM administration. Groups were tested weekly until week 4. Treatment starting at day 3 (+3) was tested only at week 4. Renal Histology was performed on kidney sections for detection of crescents formation on week 2 **(A)**. The percentage of glomeruli with crescents was evaluated in 100 glomeruli/rat on weeks 1, 2, 3, and 4 after αGBM administration **(B)**. Data are presented as mean ± SE of 8 rats/group for each time point. ^#^*p* < 0.01 increase vs. control; *p < 0.05 decrease vs. αGBM. **p < 0.01 decrease vs. αGBM. The two-way ANOVA was used.

Crescent formation in some rats was already evident 1 week after αGBM administration. This pathology was increased in the subsequent weeks (Figure 2). Treatment with AS101 before or after αGBM administration significantly ameliorated crescent formation at all time points (Figure 2B).

### AS101 Regulates IL-18, IL-1β, and Caspase-1 Activity and Ameliorates Kidney Pathology

**Figure 3:**
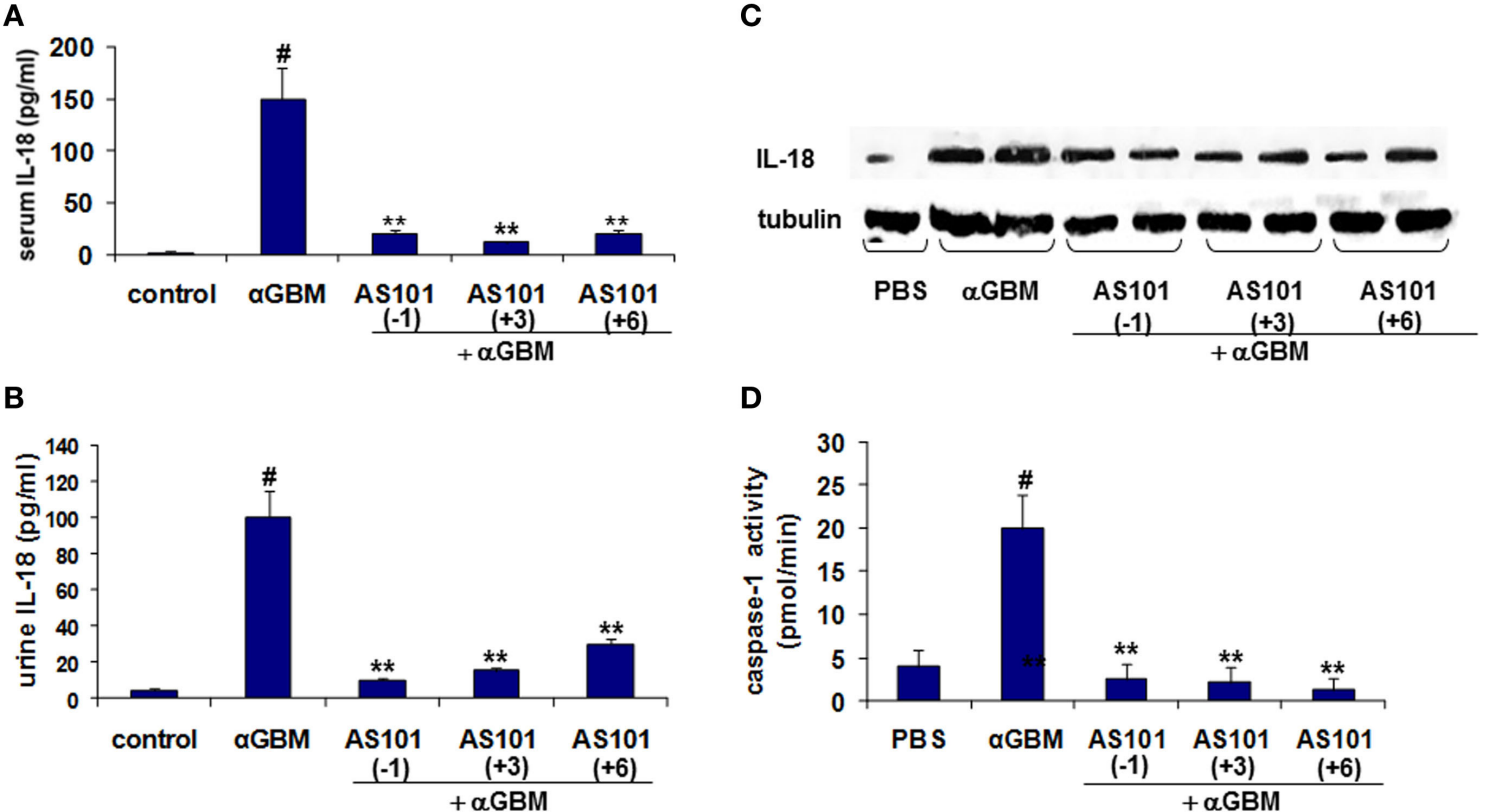
**Treatment with AS101 decreases IL-18 protein levels and glomerular caspase-1 activity in crescentic glomerulonephritis**. Treatment protocol was as described in Figure 2. Serum **(A)**, urine **(B)**, and glomerular **(C)**. IL-18 expression was evaluated 2 weeks after αGBM administration. Glomerular caspase-1 activity **(D)** was evaluated 2 weeks after αGBM administration. Data are presented as mean ± SE of 7–8 rats/group. Healthy controls (*N* = 3). ^#^*p* < 0.01 increase vs. control; **p < 0.01 decrease vs. αGBM. The one-way ANOVA was used.

It is well established that in experimental CGN IL-18 and IL-1β contribute to crescent formation and inflammatory cell recruitment (21, 22). For cytokine evaluation, we chose the 2 weeks’ time point in order to allow a reasonable time for the AS101(+6) protocol to be effective. IL-18 levels in both serum and urine were significantly increased 2 weeks after αGBM administration (Figures 3A,B). Treatment with AS101 either before or after αGBM administration significantly decreased IL-18 levels in serum as well as in urine. Moreover, glomerular IL-18 protein expression was also considerably decreased in treated rats (Figure 3C). This decrease was significant in all three regimens of AS101 administration. Importantly, the regulation of IL-18 by AS101 was positively and significantly correlated with disease severity (proteinuria) as analyzed by Pearson correlation test (Figure S1A in Supplementary Material).

IL-18 is synthesized as an inactive precursor that lacks a secretion signal sequence and is proteolytically activated by Caspase-1 (23). We therefore evaluated the effect of AS101 on caspase-1 activity in glomeruli of treated rats. Figure 3D shows a significant increase in glomerular caspase-1 activity at 2 weeks post αGBM administration, which was dramatically and significantly decreased by all three AS101 treatment regimens.

**Figure 4:**
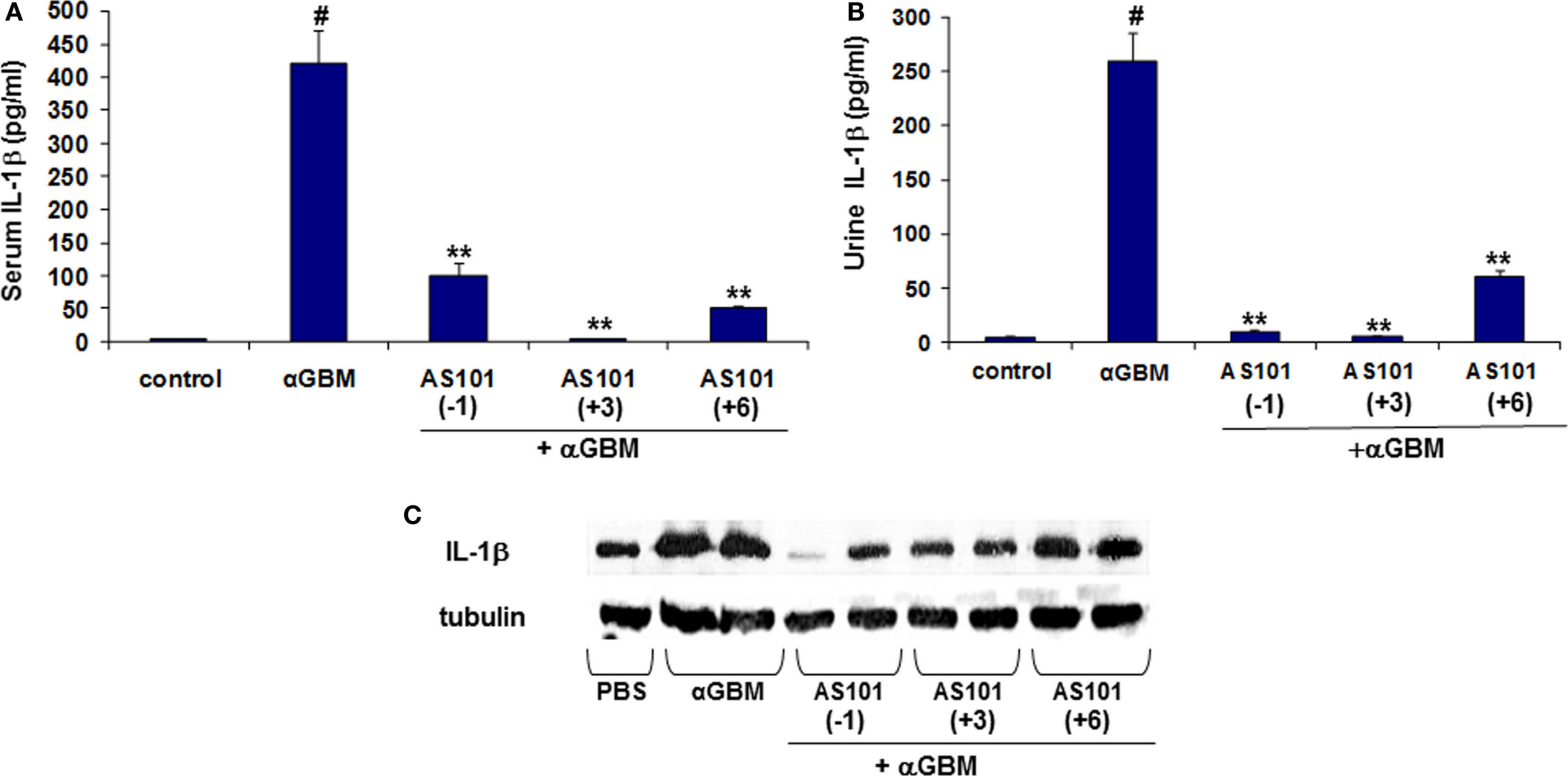
**Treatment with AS101 decreases IL-1β protein levels in crescentic glomerulonephritis**. Treatment protocol was as described in Figure 2. Serum **(A)**, urine **(B)** and glomerular **(C)** IL-1β expression were evaluated 2 weeks after αGBM administration. Data are presented as mean ± SE of 7–9 rats/group. Healthy controls (*N* = 3). ^#^*p* < 0.01 increase vs. control; **p < 0.01 decrease vs. αGBM. The one-way ANOVA was used.

These results prompted us to evaluate levels of IL-1β, an inflammatory cytokine also proteolytically activated by caspase-1 (24–26). Figure 4 shows that similar to IL-18, serum and urine levels of IL-1β were substantially and significantly increased 2 weeks after αGBM administration. Again, all three AS101 treatment regimens significantly decreased IL-1β detectable in serum and in urine at 2 weeks post αGBM administration (Figures 4A,B). Similarly, glomerular IL-1β protein was significantly decreased following AS101 treatment (Figure 4C). Importantly, both serum and urine IL-β levels were positively and significantly correlated with % glomeruli with crescent formation and with proteinuria (Figures S1B,C in Supplementary Material).

### Regulation of TNFα Expression and Caspase-3 Activity

**Figure 5:**
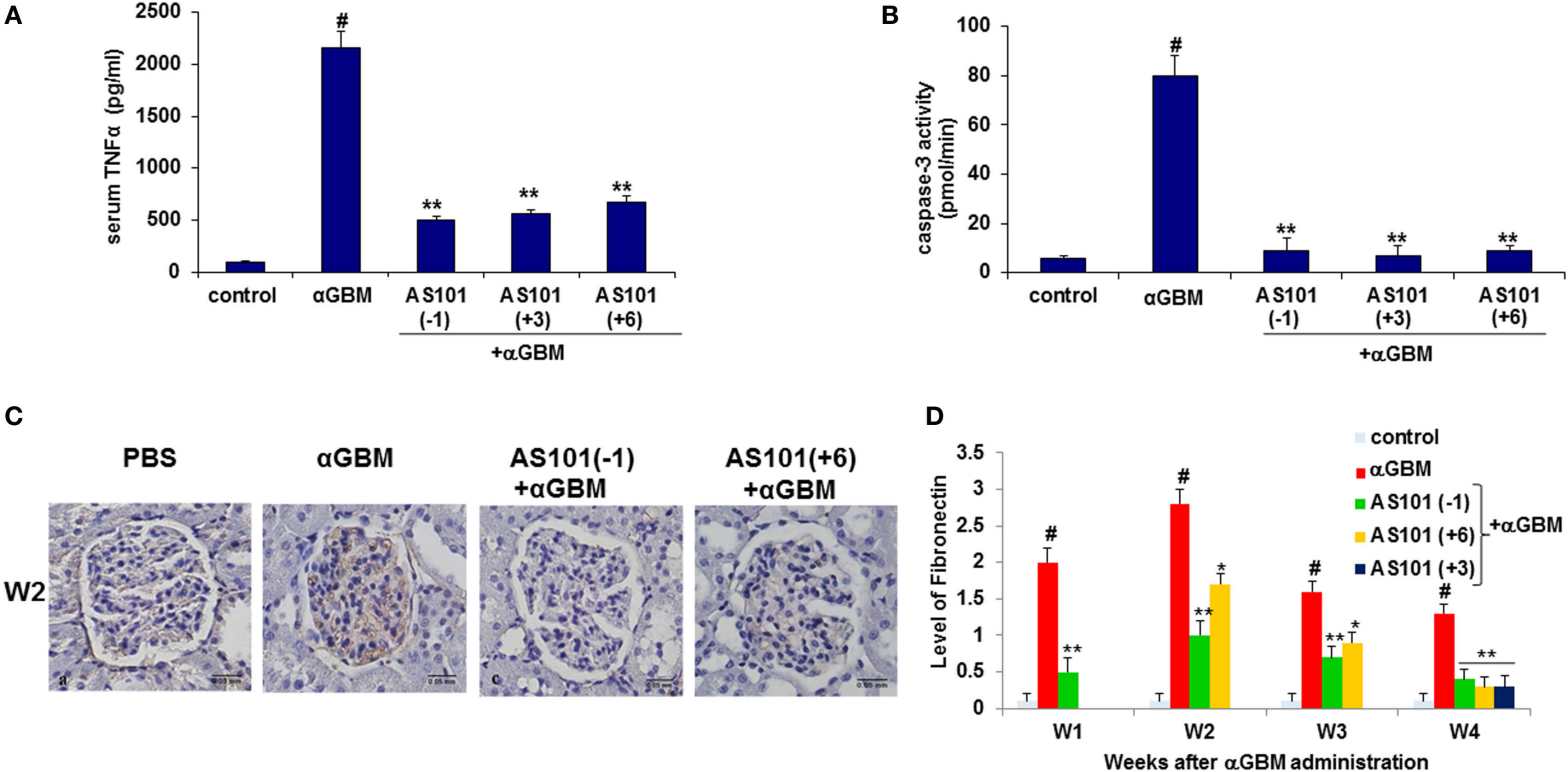
**Treatment with AS101 decreases TNFα protein levels and glomerular caspase-3 activity in crescentic glomerulonephritis**. Treatment protocol was as described in Figure 2. Serum TNFα **(A)** and glomerular caspase-3 activity **(B)** were evaluated 2 weeks after αGBM administration (*N* − 3/group). ^#^*p* < 0.01 increase vs. control; **p < 0.01 decrease vs. αGBM. The one-way ANOVA was used. Immunohistochemistry was performed on kidney sections for detection of fibronectin (FN) on week 2 **(C)**. FN expansion was evaluated on weeks 1, 2, 3, and 4 and was scored in 100 glomeruli/sample as follows: grade 0 no staining; grade 1 staining occupying up to 25% of glomerular surface area; grade 2 staining occupying 25–50% of glomerular surface area; grade 3 staining occupying more than 50% of glomerular surface area **(D)**. Data are presented as mean ± SE of five rats/group for each time point. ^#^*p* < 0.01 increase vs. control; *p < 0.01 decrease vs. αGBM. *p < 0.05 decrease vs. αGBM. The two-way ANOVA was used.

IL-18 is known to induce production of proinflammatory cytokines like TNFα and IL-1β (27). Furthermore, neutralization of endogenous IL-18 and TNF-α has been reported to reduce crescent formation, and tubulointerstitial scarring, with preservation of renal function (8, 21). Figure 5A shows that TNFα levels were strongly elevated in diseased rats, and there was a significant reduction in serum TNFα levels by all three AS101 treatment regimens. Moreover, there was a significant positive correlation between serum and urine IL-18 and IL-1β and TNFα (Figure S1D in Supplementary Material). TNFα can promote apoptosis following ligation of its receptor (28). Glomerular caspase-3 activity, the hallmark of apoptosis, was significantly increased in diseased rats and was decreased by AS101 in all treatment regimens (Figure 5B). A strong positive correlation between serum TNFα levels and glomerular caspase-3 activity supports a connection between these two parameters (Figure S1E in Supplementary Material).

### AS101 Reduces FN Accumulation in the Glomeruli

Fibronectin is a multifunctional matrix protein that increases during glomerular injury. During inflammation, both glomerular accumulation and increased synthesis of FN have been reported (29).

Figures 5C,D show a dramatic increase in FN accumulation in glomeruli of diseased rats. Treatment with AS101 significantly inhibited this process irrespective of whether AS101 was administered starting before or after αGBM administration. A strong positive correlation was found between FN accumulation and proteinuria.

### AS101 Regulates Macrophage Infiltration into the Glomeruli

**Figure 6:**
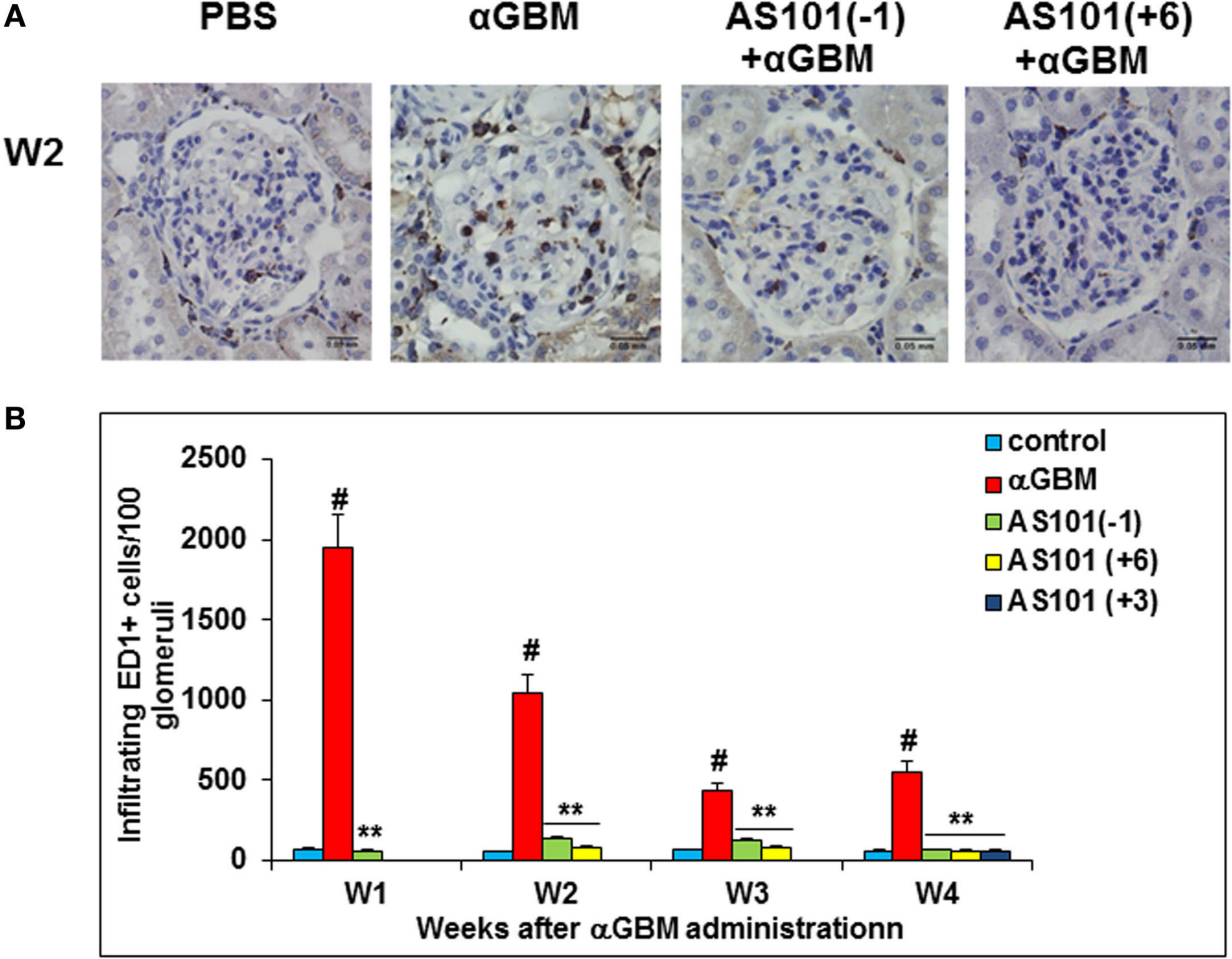
**Treatment with AS101 decreases glomerular macrophage accumulation in crescentic glomerulonephritis**. Treatment protocol was as described in Figure 2. Immunohistochemistry was performed on kidney sections for detection of glomerular ED-1 positive accumulation on week 2 **(A)**. Glomerular macrophages were counted under light microscope. One hundred glomeruli were counted/sample. Treatment starting at day 3 was examined only at week 4 **(B)**. Data are presented as mean ± SE of five rats/group for each time point. ^#^*p* < 0.01 increase vs. control; **p < 0.01 decrease vs. αGBM. The two-way ANOVA was used.

Monocyte/Macrophages play an important role in the induction of CGN and glomerular injury (5). Their accumulation has been associated with increased IL-1β and IL-18 levels in various inflammatory states (30). Immunohistochemical analysis of kidneys from αGBM-induced rats showed a significant decrease in infiltrating ED1+ cells at all time points examined, by all three regimens of AS101 administration (Figures 6A,B). Furthermore, a positive and significant correlation was found between glomerular macrophage accumulation and levels of serum and urine IL-1β (Figure S2A in Supplementary Material) and IL-18 (Figure S2B in Supplementary Material). These results are in line with the known role of these inflammatory cytokines in macrophage accumulation and suggest that AS101 might reduce glomerular macrophage accumulation by inhibiting IL-1β and IL-18 production.

**Figure 7:**
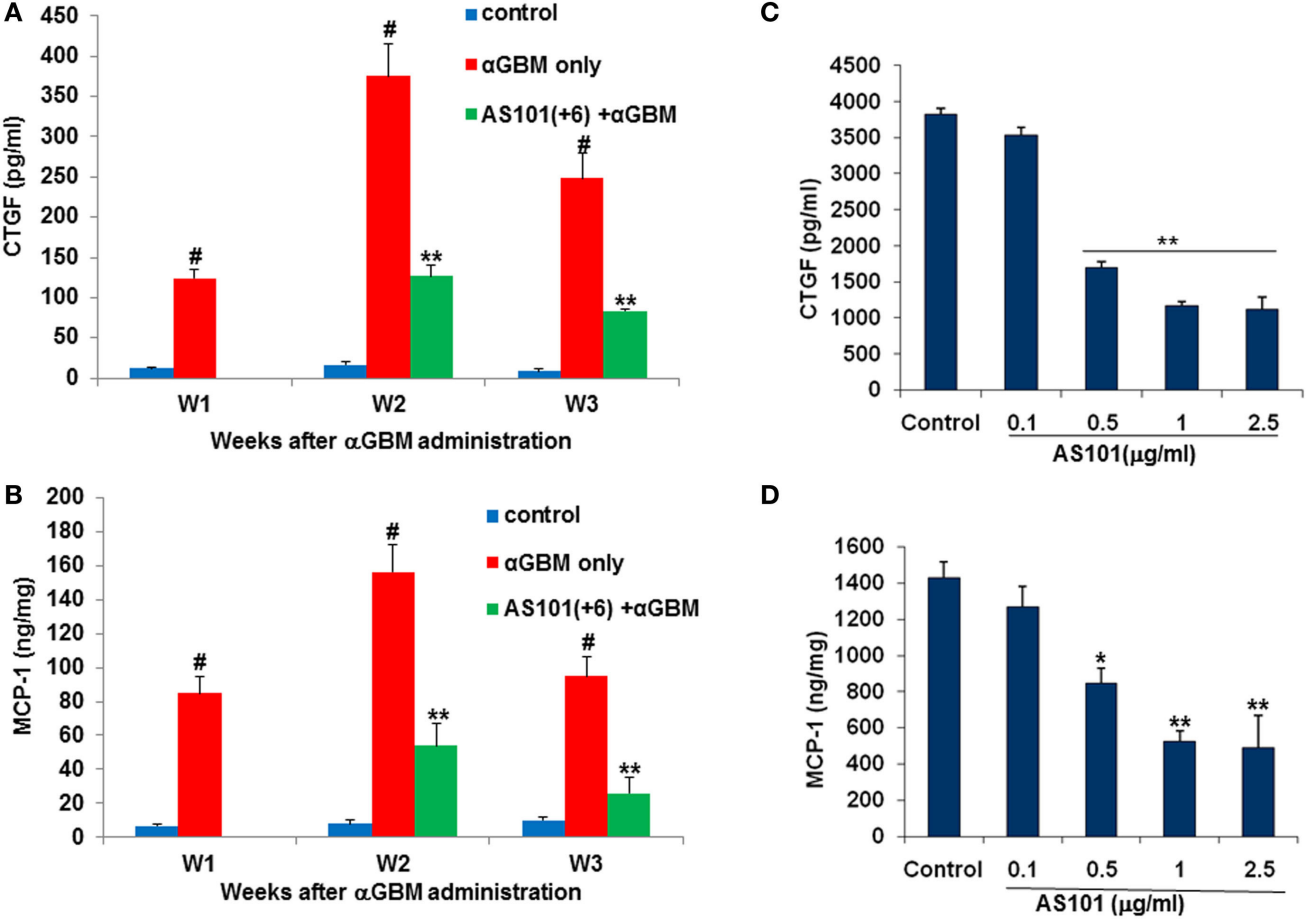
**Treatment with AS101 decreases glomerular connective tissue growth factor (CTGF) and monocyte chemoattractant protein-1 (MCP-1) protein expression in crescentic glomerulonephritis and *in vitro***. Rats were treated as follows: daily i.p. injection with PBS with no αGBM administration (control); daily injection with PBS after αGBM injection (αGBM); daily i.p. injection with AS101 (100 μg/rat) starting 6 days after αGBM administration (AS101(+6) + αGBM); Glomerular CTGF **(A)** or MCP-1 **(B)** protein expression were evaluated on weeks 1, 2, and 3 after αGBM administration. Data are presented as mean ± SE of 5 rats/group for each time point. ^#^*p* < 0.01 increase vs. control; **p < 0.01 decrease vs. αGBM. The two-way ANOVA was used. Glomerular mesangial cells were cultured on fibronectin-coated plates with or without various doses of AS101 and 10 μg/ml LPS for 48 h. Supernatants were collected and assayed for CTGF **(C)** and MCP-1 **(D)** content by ELISA. Results represent mean ± SE of three experiments. *p < 0.05 decrease vs. control; **p < 0.01 decrease vs. control. The one-way ANOVA was used.

We also examined CTGF and MCP-1, two factors involved in monocytes recruitment to inflammatory sites (31, 32) and implicated in the pathogenesis of CGN (33, 34). Both CTGF and MCP-1 were elevated in glomeruli of αGBM-induced rats at all time points examined, and both were significantly downregulated by AS101 treatment (Figures 7A,B). Expression of CTGF and MCP1 in glomeruli and their regulation by AS101 were at least in part intrinsic to the glomerular mesangial cells, as AS101 reduced production of these factors in mesangial cell cultures in a dose-dependent fashion (Figures 7C,D).

### Inactivation of VLA-4 Is Involved in the Anti-inflammatory Activity of AS101 in CGN

Very Late Antigen-4, or α4β1 integrin, is expressed mainly on monocytes, lymphocytes, and eosinophils. Blockade of VLA-4 has been shown to prevent progression of experimental CGN, but the mechanisms involved in the beneficial effects of such blockade were not elucidated. We recently showed that AS101 inactivates the VLA-4 integrin by redox modulation of vicinal thiols within the exofacial membranal side (12). Recently, another integrin, α5β1 (VLA-5), was identified as a cell membrane receptor for a parasite-associated protein in human monocytes/macrophages, leading to activation of caspase-1and IL-1β transcription and indicating that integrin engagement can lead to inflammasome activation (35). We therefore hypothesized that VLA-4 might have a role in regulating caspase-1 activity and that inactivation of VLA-4 might underlie the beneficial activity of AS101 in CGN.

**Figure 8:**
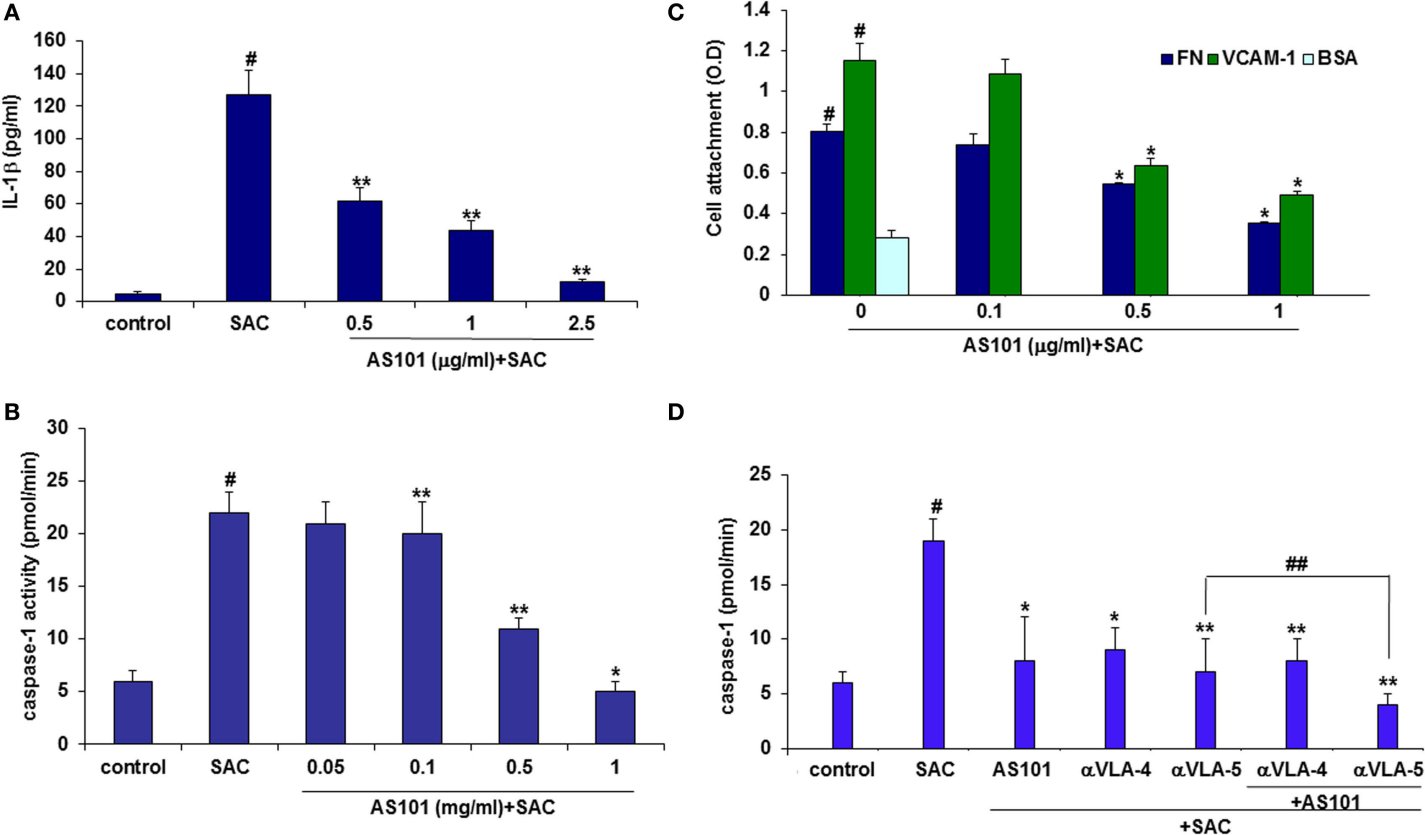
**AS101 decreases glomerular caspase-1 activity and glomerular macrophages**. IL-1β secretion *in vitro*. Caspase-1 activity is very late antigen-4 (VLA-4) dependent. Isolated glomerular macrophages were treated with or without *Staphylococcus aureus* Cowan I (SAC) (5 μg/ml) and various concentrations of AS101 at 10^6^/ml for 24 h. IL-1β **(A)** and caspase-1 activity **(B)** were evaluated. ^#^*p* < 0.01 increase vs. control, *p < 0.01 decrease vs. SAC. The one-way ANOVA was used 96-well plates were coated with VCAM-1 or fibronectin (FN) or BSA. Isolated glomerular macrophages were cultured with SAC (5 μg/ml) for 24 h. Cells were detached and cultured at 10^4^/well in triplicates in the presence or absence of various AS101 concentrations for 1 h. Non-attached cells were washed out and attached cells were quantitated by XTT **(C)**. ^#^*p* < 0.01 increase vs. BSA; *p < 0.05 decrease vs. AS101 zero (VCAM-1 or FN). The two-way ANOVA was used. Isolated glomerular macrophages were cultured as described in **(B)** in the presence or absence of AS101 (1 μg/ml), αVLA-4 (5 μg/ml), αVLA-5 (5 μg/ml), or their combinations, with or without SAC for 24 h. Caspase-1 activity was evaluated **(D)**. ^#^*p* < 0.01 increase vs. control; **p < 0.01 decrease vs. SAC; *p < 0.05 decrease vs. SAC; ^##^*p* < 0.05 decrease vs. αVLA-5 without AS101. Data are presented as mean ± SE from four different experiments. The one-way ANOVA was used.

To elucidate the role of VLA-4 inactivation by AS101 in GCN and to determine whether this activity mediates the anti-inflammatory activities of AS101, we first performed *in vitro* studies involving cultured glomerular macrophages. Figure 8 shows that glomerular macrophages secrete substantial amounts of IL-1β in response to SAC stimulation and in parallel, their caspase-1 activity is also elevated (Figures 8A,B). AS101 significantly inhibited both these activities in a dose-dependent manner. Glomerular macrophage VLA-4 activity was then evaluated by attachment to the VLA-4-specific ligand VCAM-1 and to FN while BSA served as a global negative control for cell attachment. Treatment with AS101 significantly inhibited macrophage attachment to both VCAM-1 and FN in a dose-dependent manner, implying that AS101 inhibits VLA-4 activity in macrophages (Figure 8C). Finally, we examined the role of VLA-4 and VLA-5 on caspase-1 activation and the effects of AS101 on this process. Figure 8D shows that both anti VLA-4- and VLA-5-neutralizing antibodies reduce caspase-1 activity in macrophages, implying the involvement of both integrins in caspase activation. Combined treatment of macrophages with AS101 and anti VLA-4 antibodies did not increase the extent of inhibition compared to AS101 and anti VLA-4 individually, suggesting that they both may share the same target site. In contrast, combined treatment of macrophages with anti VLA-5 antibodies and AS101 increased the extent of inhibition compared to each one individually, suggesting that they use a different binding site. Collectively, these data suggest that inhibition of VLA-4 activity by AS101 in macrophages plays an important role in caspase-1 inactivation.

### Attenuation of CGN by AS101 Is Associated with Inactivation of VLA-4 in Glomerular Macrophages

**Figure 9:**
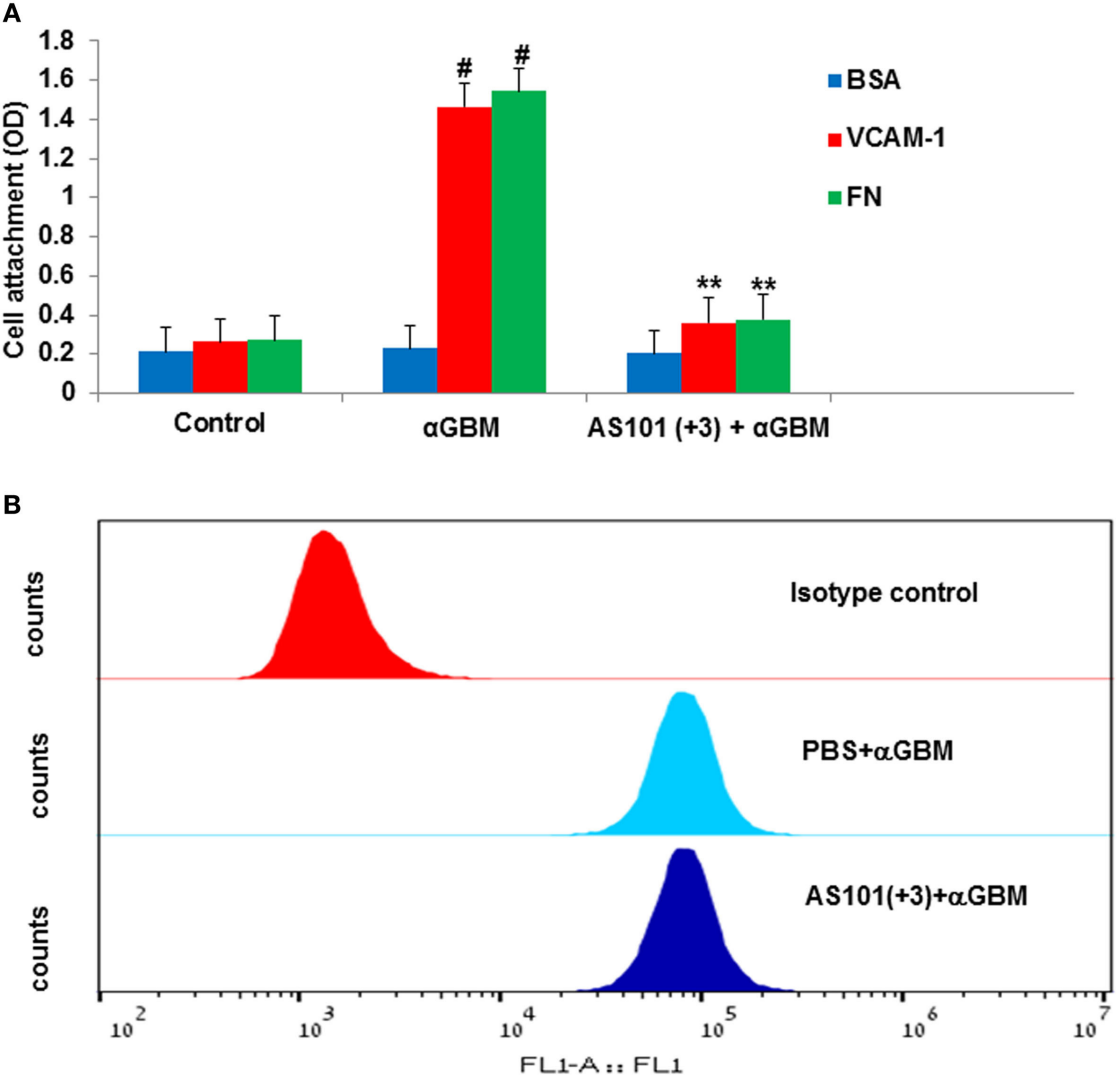
**Treatment with AS101 decreases very late antigen-4 (VLA-4) activity on glomerular macrophages**. Rats were treated as follows: daily i.p. injection with PBS with no αGBM administration (control); daily injection with PBS of αGBM-induced rats (aGBM); daily i.p. injection with AS101 (100 μg/rat) starting 3 days after αGBM administration (AS101 (+3) + αGBM); At day 14 after αGBM administration, macrophages were isolated from glomeruli of treated rats and cultured on VCAM-1, fibronectin, or BSA-coated microwells at 10^4^/well in triplicates for 1 h. Non-attached cells were washed out and the extent of the remaining attached cells was evaluated by XTT **(A)**. Data are presented as mean ± SE from four rats/group. ^#^*p* < 0.01 increase vs. control; **p < 0.01 decrease vs. αGBM. The two-way ANOVA was used **(A)**. Rats were treated with anti-glomerular basement membrane (anti-GBM) and PBS or with anti-GBM and AS101(+3). At 2 weeks after anti-GBM administration, macrophages were isolated from glomeruli of rats and evaluated for VLA-4 expression by fluorescence-activated cell sorter analysis. **(B)** Results show one representative experiment of four rats/group analyzed.

Experiments designed to measure attachment of macrophages isolated from glomeruli of CGN vs. control rats to VCAM-1 or FN revealed CGN macrophages attach more strongly to the VLA-4 ligands than do macrophages from healthy rats, suggesting a very high VLA-4 activity (Figure 9A). Treatment of rats with AS101 starting 3 days after αGBM administration (+3), significantly decreased macrophage VLA-4 activity as expressed by a prompt decrease in macrophage attachment to VCAM-1 (Figure 9A). Furthermore, the decrease in VLA-4 activity in infiltrating macrophages from AS101-treated rats derives from direct inhibition of VLA-4 activity and not merely from suppression of VLA-4 expression (Figure 9B).

These data collectively imply that inactivation of glomerular macrophage VLA-4 activity by AS101 and the consequent inactivation of VLA-induced caspase-1 activation could be causally connected to attenuation of CGN by the compound.

## Discussion

In this study, we present data showing that administration of the non-toxic small molecule tellurium compound, AS101, ameliorates crescent formation and preserves renal function in a rat model of CGN induced by anti-GBM antibodies. Our data reveal that AS101 acts by exerting antiapoptotic and VLA-4-mediated anti-inflammatory effects both *in vitro* and *in vivo* through a novel mechanism of action.

The rat model of CGN is characterized by clinical deterioration of kidney function as a result of excessive inflammatory processes followed by glomerular crescents formation and eventually glomerulosclerosis.

Apart from the role of humoral immunity in CGN, the involvement of T cells and macrophages has been long been recognized (33, 36, 37), suggesting an additional contribution of cell-mediated immunity in the pathology of the disease. Although VLA-4 inactivation by AS101 might also affect adaptive immune responses, the study focuses on the role of glomerular macrophage VLA-4 deactivation by AS101 in AS101’s beneficial effects in this disease. This is in light of numerous reports showing that macrophages are key effectors of disease progression in crescentic GN.

Our study establishes that administration of AS101 either before or after induction of CGN ameliorates renal function and crescent formation. This was associated with profound inhibition of inflammatory mediators including active caspase-1, IL-1β, IL-18, MCP-1, and CTGF, and with inhibition of FN glomerular expansion. These were accompanied by decreased glomerular infiltration of macrophages. Monocytes/macrophages play an important role in the induction of CGN and glomerular injury (5). Their accumulation has been associated with increased IL-1β and IL-18 levels in various inflammatory states (30). Moreover, IL-18 and IL-1β contribute to crescent formation and inflammatory cell recruitment (21, 22). IL-18 is known to induce the production of proinflammatory cytokines like TNFα and IL-1β (27). Importantly, neutralization of endogenous IL-18 and TNF-α has been reported to reduce crescent formation, and tubulointerstitial scarring, with preservation of renal function, in experimental CGN (8, 21). Our study shows positive and significant correlations between the decrease of these inflammatory mediators by AS101 and the amelioration increscent formation, kidney function, and macrophage recruitment. Notably, CTGF and MCP-1, which are downregulated in glomeruli of AS101-treated rats, were shown to be implicated in monocyte recruitment to inflammatory sites (9, 11) and in the pathogenesis of CGN (33, 34). The study shows a dramatic increase in FN expansion in glomeruli of diseased rats. During inflammation, both glomerular accumulation and synthesis of FN has been reported (32). Treatment with AS101 significantly inhibited this process irrespective of whether AS101 was administered starting before or after αGBM administration. There is increasing evidence that cytokines such as TNFα, IL-1β, and IL-18 play a central role in modulating endothelial function, cellular infiltration and proliferation, and extracellular matrix production (38). Furthermore, abnormal ECM accumulation plays a role in modulating the pattern of synthesis of MCP-1 by glomerular mesangial cells (39). Matrix accumulation might also play a role in maintaining monocyte infiltration after the development of ECM expansion in the subsequent phase of glomerular diseases. Expansion of ECM might be involved in the progression of glomerular diseases by manipulating the number of infiltrating monocytes and stimulation of cytokine gene expression through the induction of MCP-1 expression (39). Monocytes are involved not only in acute inflammation but also in glomerulosclerosis, a feature common to both immune and non-immune forms of progressive renal disease (40). Collectively, these data are in line with reports showing increased MCP-1 production and FN accumulation by CTGF (41, 42).

Previously, VLA-4 has been shown by two studies to prevent progression of experimental CGN (43, 44). The mechanisms of its beneficial effects were not elucidated. Nevertheless, both studies concluded that inhibition of VLA-4 activity does not affect leukocytes recruitment to the glomeruli. Our results suggest otherwise. We have recently shown that AS101 inactivates the VLA-4 integrin by specific redox modulation (12), driving a variety of beneficial biological effects in diverse preclinical and clinical studies (14). Our current study demonstrates that AS101 deactivates macrophage VLA-4 both *in vitro* and *in vivo* and presents evidence that inhibition of caspase-1 activity, associated with the anti-inflammatory activity of AS101, is dependent on VLA-4 inactivation. We speculate that the apparent discrepancy between our data and those of others, who failed to see an effect of VLA-4 blockade on macrophage recruitment, results from the unique mode of VLA-4 inactivation by AS101 as compared to neutralizing VLA-4 antibodies used by others. In fact, the possibility remains that similarly to the known phenomenon that activation of an integrin by two different ligands may exert different outcomes, its inhibition by diverse mechanisms may differentially affect cell functions. Although VCAM-1 and FN are both ligands for VLA-4, they interact with VLA-4 by a totally different mechanism (45), which might result in distinct functions. For example, the interaction between VLA on AML cells and FN results in the resistance of leukemic cells to chemotherapy, while the interaction of VLA-4 on the same cells with VCAM-1 does not (12, 46). Alternatively reduction of infiltrating macrophage could not solely depend on blocking the function of VLA-4, but may be caused by the overall anti-inflammatory effect of AS101, since AS101 can profoundly inhibit several inflammatory cytokines.

Our study is not the first demonstration that an integrin can trigger processes leading to production of inflammatory mediators. Recently, activation of the α5β1 integrin was shown to trigger macrophages to produce an extracellular burst of ATP through opening surface pannexin-1 channels that were activated by α5β1 integrin signaling in the setting of a pathogen stimulus. Subsequently, ATP delivered a critical stimulus to activate the NLRP3 inflammasome (35). Furthermore, β1 integrins have been reported to function as pathogen recognition receptors on intestinal epithelial cells to rapidly induce inflammasome-derived IL-18-mediated responses (47). Our results support and expand on these studies, and suggest for the first time that caspase-1 activity is dependent on active VLA-4. Similarly, integrins have been previously shown to induce expression of MCP-1 *via* focal adhesion kinase in mesangial cells (39). Cell adhesion to FN induced phosphorylation of FAK and MCP-1 mRNA expression. Our *in vitro* data similarly show MCP-1 and CTGF downregulation in mesangial cells cultured on FN-coated plates in the presence of AS101. In the aggregate, our data strongly support the interpretation that inactivation of VLA-4 by AS101 leads to decreased caspase-1 activity, which brings about a prompt decrease in the level of inflammatory cytokines and chemokines produced in the glomeruli of diseased rats. This in turn results in decreased ECM expansion, reducing macrophage accumulation in the glomeruli and ameliorating glomerular cell death and glomerular fibrosis, which results in preservation of kidney function.

Besides our prototype tellurium compound AS101, the investigation of therapeutic activities of other tellurium (IV) compounds is scarce in the literature, although tellurium is the fourth most abundant trace element in the human body. Our integrated results show that amelioration of the clinical status of rats with CGN can be achieved by inhibition of VLA-4 on glomerular macrophages leading to decreased caspase-1 activity followed by a significant decrease in inflammation. This regulation may be achieved using AS101, currently being tested in clinical trials and might be beneficial in the treatment of patients with CGN.

## Author Contributions

YH performed and analyzed the experiments. IS provided technical assistance and contributed to the preparation of the figures. YK, RC, and BS designed the study, discussed the results, and wrote the paper. UG provided technical assistance. All the authors discussed the results and commented on the manuscript.

## Conflict of Interest Statement

We hereby declare that no author has relationships with companies that may have a financial interest in the information contained in the manuscript. There is no interest to disclose.
