# Supplementary material for: The Small Tellurium Compound AS101 Ameliorates Rat Crescentic Glomerulonephritis: Association with Inhibition of Macrophage Caspase-1 Activity via Very Late Antigen-4 Inactivation
Source: Front Immunol. 2017 Mar 7;8:240. doi: 10.3389/fimmu.2017.00240 (PMC5339302; doi:10.3389/fimmu.2017.00240)
Supplement: Supplementary file 1 [file Image_1.PDF]

a

Suppl Fig.1

	serum IL-18	Urine IL-18
Proteinuria: Pearson correlation<br>Sig (2 tailed)<br>N	0.593<br>0.000<br>39	0.788<br>0.000<br>32

d

	Serum IL-18	urine IL-18	Serum IL-1β	urine IL-1β
TNFα:Pearson Correlation<br>sig(2-tailed)<br>N	0.934<br>0.000<br>15	0.896<br>0.006<br>12	0.842<br>0.000<br>15	0.866<br>0.000<br>12

b

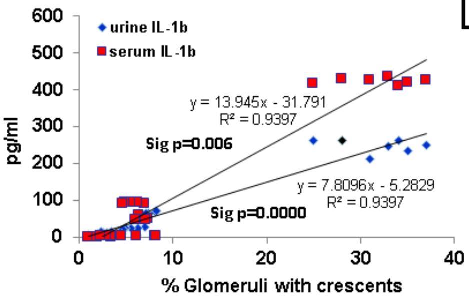

e

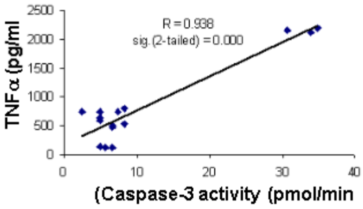

c

	Serum IL-1β	Urine IL-1β
Proteinuria: Pearson correlation<br>Sig (2tailed)<br>N	0.558<br>0.000<br>39	0.512<br>0.0003<br>32

Supplemental Figure 1. GN was induced by a single intravenous injection of αGBM (Sheep anti rat GBM) in rats pre-sensitized 5 days earlier with sheep IgG, as described in Methods. Experimental groups were as follows: daily I.P. injection with PBS without αGBM administration (negative control); daily injection with PBS of αGBM induced rats (positive control) and three treatment groups of daily I.P. injections with AS101 (100 µg/rat) starting 1 day before (–1), 3 days after (+3) or 6 days after (+6) the αGBM administration. Serum and urine IL-18 levels were evaluated 2 weeks after αGBM administration. Pearson correlation test was applied between proteinuria, serum and urine IL-18. Samples from both treated and untreated groups were used for analysis. (a). Pearson correlation test was applied between % glomeruli with crescents, serum and urine IL-1β. Samples from both treated and untreated groups were used for analysis. (b). Pearson correlation test was applied between proteinuria, serum and urine IL-1β. Samples from both treated and untreated groups were used for analysis. (c). Pearson correlation test was applied between Serum TNFα, serum and urine IL-1β, and serum and urine IL-18 (d). Pearson correlation test was applied between Serum TNFα and glomerular caspase-3 activity. Samples from both treated and untreated groups were used for analysis.(e).
