# Supplementary material for: The Small Tellurium Compound AS101 Ameliorates Rat Crescentic Glomerulonephritis: Association with Inhibition of Macrophage Caspase-1 Activity via Very Late Antigen-4 Inactivation
Source: Front Immunol. 2017 Mar 7;8:240. doi: 10.3389/fimmu.2017.00240 (PMC5339302; doi:10.3389/fimmu.2017.00240)
Supplement: Supplementary file 2 [file Image_2.PDF]

Suppl Fig. 2

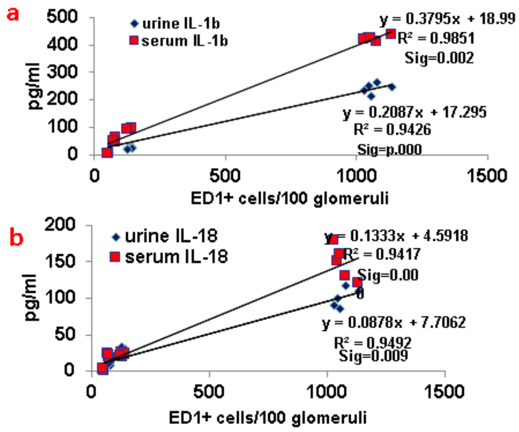

Supplemental Figure 2. GN was induced as described in Fig. 1. Treatment protocol was as described in Fig. 1. Pearson correlation test was applied between urine and serum IL-1β and the number of ED-1+ cells/100 glomeruli (a) and between urine and serum IL-18 and the number of ED-1+ cells/100 glomeruli (b). Samples from both treated and untreated groups were used for analysis.
